# Severe lymphopenia as a prognostic factor in rectal cancer patients receiving adjuvant chemoradiotherapy: a retrospective study

**DOI:** 10.1038/s41598-023-34145-4

**Published:** 2023-05-09

**Authors:** Shuang Li, Weiping Yao, Ruiqi Liu, Yanwei Lu, Haibo Zhang, Xiaodong Liang

**Affiliations:** 1 Cancer Center, Department of Radiation Oncology, Zhejiang Provincial People’s Hospital, Jinzhou Medical University, Jinzhou, 121001 Liaoning China; 2 Cancer Center, Department of Radiation Oncology, Zhejiang Provincial People’s Hospital, Affiliated People’s Hospital, Hangzhou Medical College, Hangzhou, 310014 Zhejiang China

**Keywords:** Cancer models, Cancer prevention, Cancer screening, Cancer therapy, Cancer, Diseases, Health care, Oncology, Risk factors

## Abstract

The relationship between total lymphocyte counts (TLCs) and survival is not well documented in rectal cancer. This study aimed to investigate the association between TLCs and disease-free survival (DFS) and identify factors associated with lymphopenia in locally advanced rectal cancer patients receiving chemoradiotherapy. Thirty-six patients with locally advanced rectal cancer were retrospectively analyzed. TLCs were evaluated before surgery (pre-S), before radiotherapy (pre-RT), and during concurrent chemoradiotherapy (CCRT). The relationship between TLCs and DFS was analyzed by univariate and multivariate analysis. Potential clinical factors associated with lymphopenia were also evaluated. Median TLC declined significantly during radiotherapy. Severe lymphopenia during CCRT was significantly associated with poorer DFS on Kaplan–Meier analysis (p = 0.01), univariate regression analysis (p = 0.036), and multivariate regression analysis (p = 0.038). Pre-S TLCs (p = 0.009) and pre-RT TLCs (p = 0.042) were significantly associated with severe lymphopenia on univariate regression analysis; however, only pre-S TLCs (p = 0.026) were significantly associated with severe lymphopenia on multivariate regression analysis. Severe lymphopenia was a predictor of poorer DFS in patients with locally advanced rectal cancer receiving adjuvant chemoradiotherapy. Pre-S TLCs were predictors of severe lymphopenia. Further study is warranted to reduce the rate of severe lymphopenia.

## Introduction

Colorectal cancer is the fourth most common cancer and the second leading cause of cancer death around the world^1^. Postoperative concurrent chemoradiotherapy (CCRT) can improve local control, recurrent-free survival and overall survival (OS) for locally advanced rectal cancer^2^. Preoperative CCRT results in a higher local control rate with lower toxicities, compared with postoperative CCRT based on well-designed randomized trials^3,4^. And preoperative CCRT has been accepted as the standard treatment for locally advanced rectal cancer worldwide. However, some patients were diagnosed with early rectal cancers before surgery and were found to be with locally advanced diseases after operation. In addition, some patients refused to receive preoperative CCRT. Surgery followed by CCRT is still a choice for locally advanced rectal cancer patients in clinical practice.

The immune system has been recognized as an important factor for cancer treatment. Lymphocytes play a central role in antitumor immunity. Radiotherapy can activate the immune system and promote abscopal response in combination with immune checkpoint inhibitors^5^. However, radiotherapy can reduce lymphocyte number and may thus have negative influence on survival outcomes at the same time. The level of host lymphocytes has been reported to be associated with treatment outcomes in advanced rectal cancer^6^. Kitayama et al.^7^ reported that higher circulating lymphocyte ratio is associated with good response to preoperative CCRT in patients with locally advanced rectal cancer. Heo et al.^8^ found that higher sustaining lymphocyte ratio at 4 weeks during CCRT was associated with better pathologic complete response. Moreover, pretreatment lymphopenia and posttreatment lymphopenia have been verified to be associated with prognosis in patients with lung cancer, esophageal cancer, and cervical cancer^9–11^.

Postoperative CCRT can improve local control, recurrent-free survival, and OS for locally advanced rectal cancer^2,12^. Postoperative CCRT is the standard treatment in patients with locally advanced rectal cancers who do not receive CCRT before surgery (pre-S). However, the relationship between total lymphocyte counts (TLCs) and survival outcomes has not been documented in patients with locally advanced rectal cancer undergoing operation followed by adjuvant CCRT and chemotherapy.

In this retrospective study, we examined the number of circulating lymphocytes before and during treatments and assessed their possible association with disease-free survival (DFS) in patients with locally advanced rectal cancer treated with surgery and postoperative CCRT.

## Materials and methods

### Patient selection

With approval of the Ethic Committee of Zhejiang Provincial People’s Hospital, patients were retrospectively identified at our institution between August 2013 and September 2017.The following eligibility criteria were met: newly diagnosed locally advanced rectal adenocarcinoma (T3–T4-N0 or T1–T4-N1–N2), Eastern Cooperative Oncology Group performance status of 0–1, baseline complete blood counts performed, available baseline carcinoembryonic antigen (CEA), received operation followed by postoperative CCRT at our institution, total mesolectal excision (TME) performed with R0 resection, and at least 3 weekly blood counts documented during CCRT.

### Data collection

Clinical characteristics, treatment characteristics, and data of potential prognostic factors were collected from the electronic medical record at our institution. The TLCs before surgery (pre-S TLCs) were obtained from blood samples collected 0–7 days pre-S. The TLCs before radiotherapy (pre-RT TLCs) were obtained from blood samples collected 0–7 days before the start date of adjuvant CCRT. All of the blood data during the period of CCRT were collected weekly in each patient. Toxicity on lymphocyte was graded according to the National Cancer Institute’s Common Terminology Criteria for Adverse Events version 4.0. Severe lymphopenia was defined as grade 3–4 lymphopenia (a nadir of < 0.5 × 10^9^ cells/L) during CCRT (weeks 1 to 5). The remaining patients were classified as without severe lymphopenia, while their TLCs were never < 0.5 × 10^9^ cells/L during CCRT.

### Statistical analysis

Baseline characteristics of patients were summarized using descriptive statistics. Chi-squared test or Fischer’s exact test was used for proportional comparison. Wilcoxon signed rank test for paired samples was used to compare pre-S TLCs with pre-RT TLCs. Friedman test was used to compare TLCs at different time points during CCRT. OS was calculated from the date of surgery to the date of death (all-cause mortality) or censored at the date of the last follow-up. DFS was calculated from the date of surgery to the date of first radiographic progression or recurrence or censored at the date of the last follow-up. The primary statistical end point was DFS. Kaplan–Meier curves were generated, and survival outcomes were compared using the log-rank test. The potential prognostic factors of DFS were analyzed using the univariate Cox regression model, including age, sex, CEA, pre-S TLCs, pre-RT TLCs, and with or without severe lymphopenia. All of the variables were dichotomized. Age, CEA, pre-S TLCs, and pre-RT TLCs were dichotomized at the median level. Candidate prognostic factors with a p value < 0.1 in univariate analysis were subsequently included in the multivariate analysis. The multivariate Cox proportional hazards regression model was used to evaluate hazard ratios, adjusting for the other factors. Clinical and treatment characteristics associated with severe lymphopenia were evaluated by univariate and multivariate logistic regression analysis accordingly.

All statistical tests were performed using the Statistical Package for the Social Sciences software package (SPSS) version 17.0 (SPSS Inc., Chicago, IL), and two-sided p value < 0.05 was considered to be statistically significant.

### Ethics statement

The research titled “A retrospective study of the relationship between lymphocyte number/subpopulation distribution and the outcome and prognosis of patients with rectal cancer during radiotherapy and chemotherapy” (2020QT152) has obtained human research ethics approval from the Ethics Committee of Zhejiang Provincial People’s Hospital. Informed consent was waived by the Ethics Committee of Zhejiang Provincial People’s Hospital as study being retrospective. The author has conducted the research as a member of a project or course approved by the Ethics.

### Ethics approval and consent to participate

This study was conducted in accordance with the Helsinki Declaration II and was approved by the Institutional Review Boards of Zhejiang Provincial People’s Hospital.

## Results

### Patient and treatment characteristics

All patients with rectal cancer were recommended for postoperative chemoradiotherapy when they were pathologically staged with T3–T4-N0 or T1–T4-N1–N2 in our hospital. A total of 41 patients with locally advanced rectal cancer underwent surgery followed by postoperative CCRT and postoperative chemotherapy at Zhejiang Provincial People’s Hospital during the period. Among them, 36 cases met the inclusion criteria, and all of whom were included in this study. The median age was 60.5 years (range 34–76 years); 23 (63.89%) patients were males and 13 (36.11%) were females. Four patients were pathologically diagnosed with stage T2, 22 patients with stage T3, and 10 patients with stage T4, respectively. Eleven patients were pathologically diagnosed to have node-negative cancer (stage II), and 25 patients were diagnosed to have node-positive cancer (stage III). All of the patients received adjuvant CCRT following 2 cycles of chemotherapy and followed by median 5 cycles of capecitabine/oxaliplatin every 3 weeks or median 7 cycles of fluorouracil/folinic acid/oxaliplatin every 2 weeks. Infectious diseases were not documented during adjuvant CCRT, and severe infectious diseases were not documented within 1 year from the date of surgery. For radiotherapy, clinical target volume (CTV) included the tumor bed with a margin of 2–3 cm, presacral lymph nodes, and internal iliac lymph nodes. The external iliac lymph nodes and inguinal lymph nodes were also included in some high-risk patients. CTV_boost_ comprised the tumor bed with a margin of 2–3 cm. A dose of 45 Gy was administered to planning target volume (PTV), and a dose of 50 Gy was administered to PTV_boost_ in 25 fractions using a simultaneous integrated boost intensity-modulated radiotherapy technique. Capecitabine was administered concurrently with radiotherapy at a dose of 1650 mg/m2/day (5 or 7 days every week). All of the included patients completed the planned CCRT schedule without treatment interruption of more than 3 days.

The baseline characteristics of the patients are listed in Table 1. Thirty patients were still alive after the median follow-up time of 34 months. Six patients had died on the last follow-up date (December 31, 2018). All of them died from disease progression. Twelve patients had progressive disease during follow-up. Only one patient had locally relapsed disease. All of the other patients suffered from distant metastases without local failure, six with lung metastases, two with liver metastases, two with wide spread metastases, and one with brain metastases.

**Table 1:** Patient characteristics.

Characteristics	
Number of patients	36
Median age (range)	60.5 (34–76)
Sex	
Male	23 (63.89%)
Female	13 (36.11%)
T stage	
T2	4 (11.11%)
T3	22 (61.11%)
T4	10 (27.78%)
N stage	
N−	11 (30.56%)
N+	25 (69.44%)
CEA (ng/mL)	
≤ 4.0	17 (47.22%)
> 4.0	19 (52.78%)

*N* lymph node, *N*+ lymph node positive, *N− *lymph node negative, *CEA* carcino-embryonic antigen.

### Total lymphocyte count change during treatments

**Figure 1:**
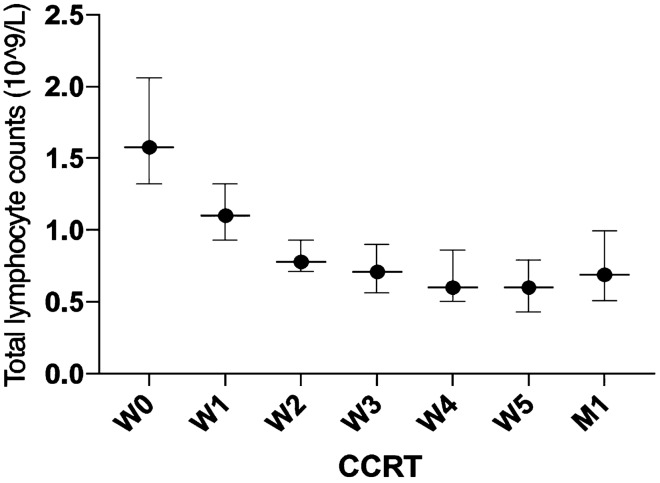
Total lymphocyte counts declined over time during concurrent chemoradiotherapy (CCRT) and restored partly 1 month after the completion of CCRT.

The median pre-S TLC was 1.57 (range 0.74–3.24) × 10^9^ cells/L, and the median pre-RT TLC was 1.58 × 10^9^ (range 0.87–3.50) cells/L in the study cohort. There was no significant difference between the median pre-S and the median pre-RT TLCs (p = 0.658). Prior to surgery, 23 patients (63.89%) had a normal TLC, 11 patients (30.56%) had grade 1 lymphopenia, 2 patients (5.56%) had grade 2 lymphopenia, and no patient had grade 3–4 lymphopenia. Twenty-four, seven, and five patients had grade 0, grade 1, and grade 2 lymphopenia before CCRT, respectively. Fifteen patients (41.67%) and 21 patients (58.33%) experienced grade 2 and grade 3 lymphopenia during CCRT. No patient had grade 0, grade 1, or grade 4 lymphopenia during CCRT. A total of 26 patients had complete data of weekly TLCs and TLCs 1 month after the completion of CCRT. The median pre-RT TLC was 1.59 (range, 0.87–3.5) × 10^9^ cells/L, and the median TLC declined significantly to 1.10 (range, 0.60–2.04), 0.78 (range 0.56–1.29), 0.71 (range, 0.29–1.25), 0.60 (range 0.25–1.23), and 0.60 (range 0.28–1.35) × 10^9^ cells/L from week 1 (W1) to week 5 (W5) during CCRT, respectively, and the median TLC was restored significantly to 0.69 (range 0.30–2.24) × 10^9^ cells/L 1 month after the completion of CCRT (Fig. 1). There were significant differences between TLCs at W0 and W1 (p = 0.001), W0 and W2 (p < 0.001), W0 and W3 (p < 0.001), W0 and W4 (p < 0.001), W0 and W5 (p < 0.001), W1 and W2 (p < 0.001), W2 and W3 (p = 0.043), and W5 and M1 (p = 0.031). No significant difference was observed between TLCs at W3 and W4 (p = 0.237) and W4 and W5 (p = 0.241).

### Patient and treatment characteristics associated with survival outcomes

**Figure 2:**
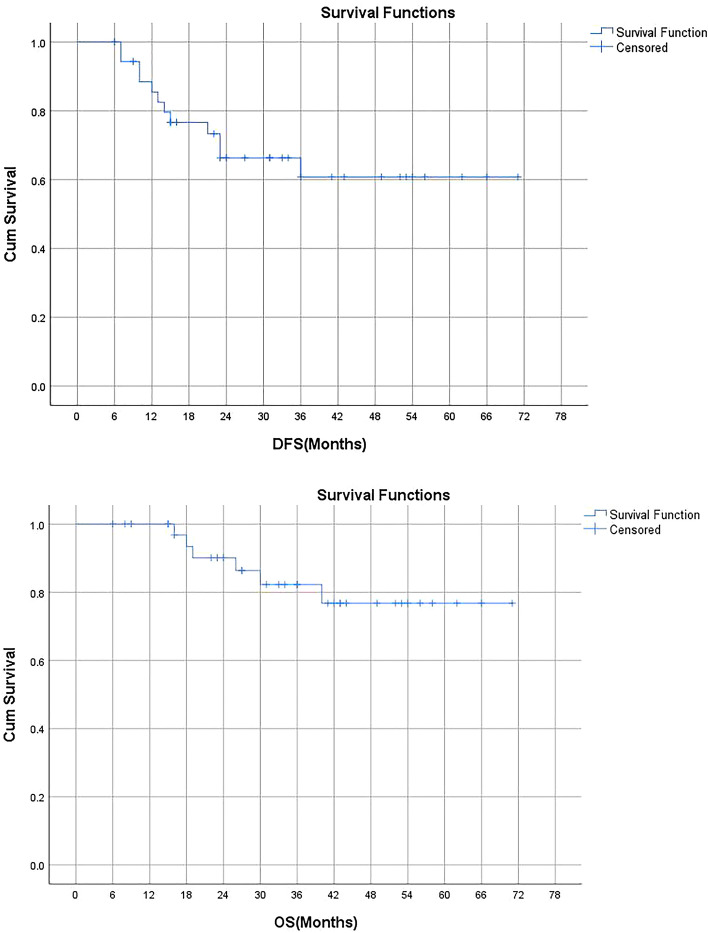
Kaplan–Meier curves showing disease-free survival (DFS) and overall survival (OS) in all patients included.

**Figure 3:**
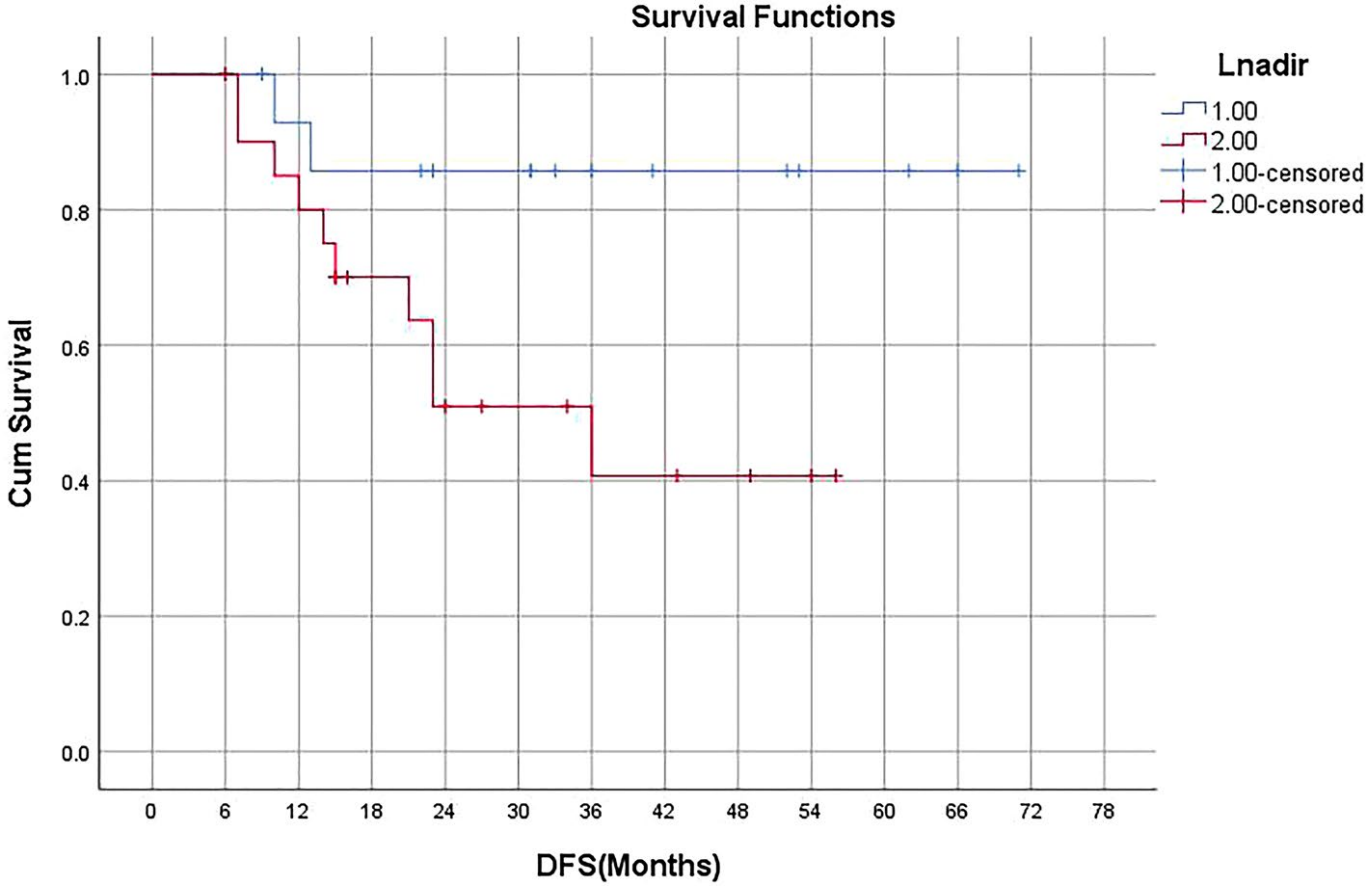
Kaplan–Meier curves showing DFS in patients without (group 1) or with severe lymphopenia (group 2) during CCRT.

**Figure 4:**
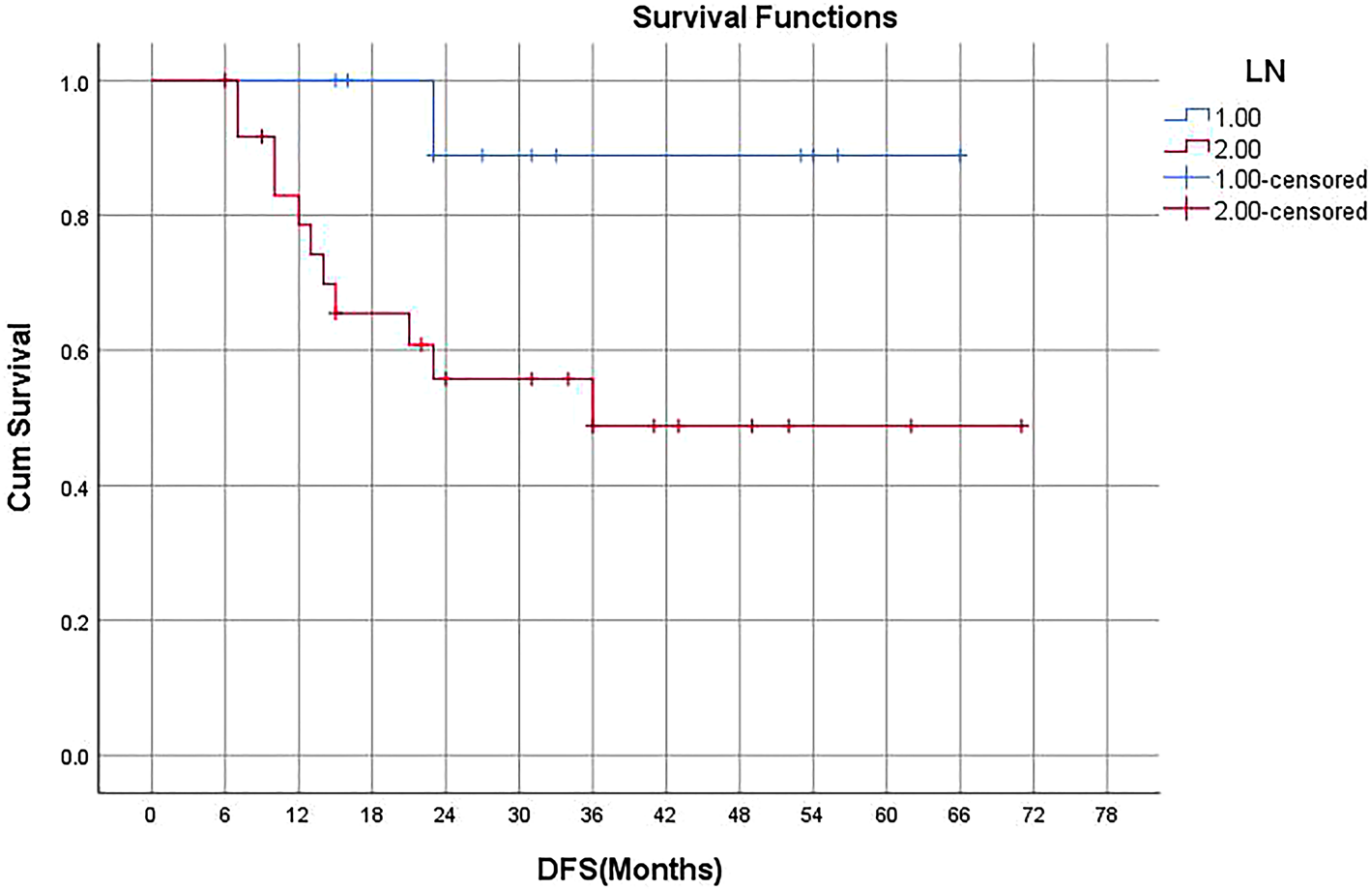
Kaplan–Meier curves showing DFS in patients without (group 1) or with positive lymph node (group 2).

DFS and OS curves of the whole cohort are shown in Fig. 2. Neither median DFS nor median OS had been achieved. Severe lymphopenia during CCRT was significantly associated with poorer DFS on Kaplan–Meier analysis (p = 0.01, detailed in Fig. 3), univariate regression analysis, and multivariate regression analysis (detailed in Table 2). Median DFS was 36 months among patients with severe lymphopenia, and median DFS had not been achieved in patients without lymphopenia (Fig. 3). Positive node was marginally significantly associated with worse DFS on Kaplan–Meier analysis (p = 0.052, detailed in Fig. 4), univariate analysis (p = 0.091), and multivariate analysis (p = 0.097, detailed in Table 2).

**Table 2:** Univariate and Multivariate analysis for variables associated with DFS.

Clinical factors	Univariate analysis			Multivariable analysis		
	HR	95% CI	p	HR	95% CI	p
Age (< 60.5 vs > 60.5)	2.572	0.247–2.658	0.729			
Sex (female vs male)	0.608	0.160–2.307	0.465			
N status (N + vs N−)	5.911	0.755–46.298	0.091	700	0.728–44.623	0.097
CEA (> 4.0 vs ≤ 4.0)	1.995	0.582–6.838	0.272			
Pre-S TLCs (low vs high)	3.052	0.809–11.517	0.100			
Pre-RT TLCs (low vs high)	2.051	0.598–7.033	0.253			
Severe lymphopenia (y vs n)	9.103	1.160–71.423	0.036	904	1.132–70.001	0.038

*HR* hazard ratio, *CI* confidence interval, *N* lymph node, *N*+ lymph node positive, *N− *lymph node negative, *CEA* carcino-embryonic antigen, *pre-S TLCs* total lymphocyte counts before surgery, *pre-RT TLCs* total lymphocyte counts before concurrent chemoradiotherapy, *y* yes, *n* no.

### Patient and treatment factors associated with severe lymphopenia

We succeedingly evaluated the association between clinical characteristics and severe lymphopenia. Pre-S TLCs were significantly associated with severe lymphopenia on Fischer’s exact test, univariate regression analysis, and multivariate regression analysis (detailed in Tables 3, 4). There was a significant difference between pre-RT TLCs and severe lymphopenia on Fischer’s exact test and univariate regression analysis (detailed in Tables 3, 4). However, no significant relationship between pre-RT TLCs and severe lymphopenia was verified on multivariate regression analysis (detailed in Table 3). None of the other clinical characteristics was found to be associated with severe lymphopenia. Additionally, none of the clinical characteristics was associated with pre-S TLCs, including age, sex, nodal status, and CEA.

**Table 3:** Univariate and Multivariate analysis for variables associated with severe lymphopenia.

Clinical factors	Univariate analysis			Multivariable analysis		
	HR	95% CI	p	HR	95% CI	p
Age (≤ 60 vs > 60)	1.257	0.333–4.742	0.735			
Sex (female vs male)	2.062	0.492–8.654	0.322			
N status (N+ vs N−)	1.250	0.299–5.230	0.760			
CEA (> 4.0 vs ≤ 4.0)	0.606	0.158–2.319	0.464			
Pre-S TLCs (low vs high)	8.000	1.686–37.981	0.009	6.246	11.248–31.257	0.026
Pre-RT TLCs (low vs high)	4.469	1.054–18.938	0.042	3.025	0.624–14.662	0.169

*N* lymph node, *N*+ lymph node positive, *N− *lymph node negative, *CEA* carcino-embryonic antigen, *pre-S TLCs* total lymphocyte counts before surgery, *pre-RT TLCs* total lymphocyte counts before concurrent chemoradiotherapy.

**Table 4:** Chi-square test or Fischer exact test for clinical variables associated with severe lymphopenia.

Clinical factors	Without severe lymphopenia	With severe lymphopenia	p value
Number of patients	16	20	
Age			1.000
≤ 60	8 (44.4%)	10 (55.6%)	
> 60	7 (38.9%)	11 (61.1%)	
Sex			0.484
Male	11 (47.8%)	12 (52.2%)	
Female	4 (30.8%)	9 (69.2%)	
N stage			1.000
N−	5 (45.5%)	6 (54.5%)	
N+	10 (40.0%)	15 (60.0%)	
CEA			0.516
≤ 4.0	6 (35.3%)	11 (64.7%)	
> 4.0	9 (47.4%)	10 (52.6%)	
Pre-S TLC			0.008
≥ 1.57	12 (63.2%)	7 (36.8%)	
< 1.57	3 (17.6%)	14 (82.4%)	
Pre-RT TLC			0.049
≥ 1.58	11 (57.9%)	8 (42.1%)	
< 1.58	4 (23.5%)	13 (76.5%)	

This project is a retrospective study and has been approved by the Ethics Committee of Zhejiang Provincial People’s Hospital with the approval number 2020QT152.Informed consent was waived by the Ethics Committee of Zhejiang Provincial People’s Hospital as study being retrospective. The author has conducted the research as a member of a project or course approved by the Ethics Committee.

## Discussion

Higher circulating lymphocyte ratio before and during treatment had been shown to be associated with good response to preoperative CCRT in patients with locally advanced rectal cancer^7^. However, no previous study has verified the relationship between lymphopenia and survival outcomes in patients with locally advanced rectal cancer receiving postoperative chemoradiotherapy. This retrospective study found that severe lymphopenia during CCRT was significantly associated with shorter DFS in patients with locally advanced rectal cancer receiving surgery and adjuvant chemoradiotherapy. Severe lymphopenia can be used as a useful prognostic factor for locally advanced rectal cancer. Furthermore, pre-S TLCs were associated with severe lymphopenia in the present study. We suggest that severe lymphopenia was the result of combined effects of surgery, radiotherapy, chemotherapy, and clinical characteristics.

Treatment-related lymphopenia has been recognized as an important independent predictor of survival outcomes in patients with cancers treated with radiotherapy in recent years. Tang et al.^9^ reported that lower TLCs were associated with shorter event-free survival and OS in patients with non-small-cell lung cancer receiving definitive radiotherapy. Cho et al.^13^ demonstrated that TLC nadir during treatment and TLCs posttreatment were considered the predictors of progression-free survival and OS in limited-stage small-cell lung cancer treated with radiotherapy and chemotherapy. Sakurai et al.^14^ also reported that lymphocyte count is possibly one of the early predictors for survival in patients with esophageal cancer. The above studies are in line with our findings. To our knowledge, this is the first study that identified the association between treatment-related lymphopenia and DFS in patients with locally advanced rectal cancer receiving postoperative chemoradiotherapy.

Pretreatment lymphopenia was also found to be significantly associated with shorter PFS and poorer OS in patients with advanced colorectal cancer receiving chemotherapy^6^. Higher pretreatment lymphocyte ratio was significantly associated with better DFS and OS in locally advanced rectal cancer^7^. Furthermore, Choi et al.^11^ demonstrated that initial TLCs were associated with progression-free survival in cervical cancer treated with CCRT. However, treatment-related lymphopenia was not investigated in the above studies. In contrast, we found that initial TLCs were not associated with DFS and that severe lymphopenia was the only independent prognostic factor in the present study. Several studies had evaluated the association between TLCs and survival outcomes in patients treated with CCRT regarding both initial TLCs and posttreatment TLCs. Tang et al.^9^ reported that TLCs before treatment were marginally significantly associated with OS on univariate analysis in patients with non-small-cell lung cancer (p = 0.09). However, lymphocyte nadir was the only significant prognostic factor for event-free survival and OS on multivariate regression analysis. Similarly, Cho et al.^13^ found that initial TLCs were significantly associated with OS on univariate analysis but not on multivariate analysis in limited-stage small-cell lung cancer patients. Additionally, treatment-related lymphopenia but not initial lymphopenia was found to be associated with survival in patients with resected pancreatic cancer^15^. These studies have consistent conclusions as ours, and the present study supports the existing evidence that treatment-related lymphopenia is a more robust prognostic factor of survival outcomes compared with initial TLCs in patients with cancer treated with radiotherapy.

TLCs declined significantly from the beginning to the end of CCRT in this study. Moreover, there was no significant difference between pre-S TLCs and pre-RT TLCs, which suggests that surgery and chemotherapy may have less profound effect on TLCs. Campian et al.^16^ found that neither neoadjuvant chemotherapy nor different regimens had influence on TLCs in patients with non-small-cell lung cancer receiving CCRT. Wild et al.^17^ found that neoadjuvant chemotherapy had no effect on the TLCs in patients with pancreatic cancer receiving definitive CCRT. Balmanoukian et al.^15^ found that most patients had normal TLCs following surgery, while TLCs dropped significantly during adjuvant CCRT in patients with pancreatic cancer. These studies, in line with ours, demonstrate that radiotherapy has a more profound effect than surgery and chemotherapy on TLCs since lymphocytes has high sensitivity to radiotherapy.

However, we found that pre-S TLCs were significantly associated with severe lymphopenia, and pre-RT TLCs were marginally significantly associated with severe lymphopenia during CCRT in the present study. Similarly, Campian et al.^18^ reported that initial lymphopenia was associated with severe treatment-related lymphopenia after CCRT in patients with head and neck cancers. Taken altogether, initial TLCs may have an impact on severe lymphopenia, while radiotherapy is not the single cause of severe lymphopenia. Severe lymphopenia is the result of combined effects of surgery, radiotherapy, chemotherapy, and patient characteristics, although radiotherapy is a profound factor for the reduction of TLCs. Hence, it is reasonable that initial TLC was considered a predictor of survival outcomes in some of the abovementioned studies.

Six patients had died, and the shortest survival time is 18 months. All of them died of disease progression, and no patient died of infection. Patients with lymphopenia are prone to be infected; however, lymphopenia-induced infection is not the cause of poorer DFS in the present study. We suggest that patients with lymphopenia may have a less robust immune system against cancer, which may result in a poorer DFS. Lymphopenia may be not only a prognostic factor but also a causal factor of poorer survival. Davuluri et al.^10^ found that proton therapy could reduce total body irradiation dose and decrease the incidence of grade 4 lymphopenia compared with photon radiotherapy. Proton therapy was associated with better OS on univariate analysis but not on multivariate analysis. It is possible that reducing the rate of severe lymphopenia could improve survival outcomes, and further study is warranted. Similarly, stereotactic body radiation therapy decreased the severity of lymphopenia in patients with locally advanced pancreatic cancer compared with conventional CCRT^19^. Lymphocyte-sparing technique should be further investigated, while radiotherapy is one of the most profound factors that have an impact on severe lymphopenia.

Only one local failure was identified in these patient cohorts. Distant metastases were the main failure, which was in accordance with the literature in the TME era^3,4^. Positive lymph node was defined as a prognostic factor and was included in tumor-node-metastasis staging system^20^. However, positive lymph nodes (stage III) were associated with inferior DFS with marginal significance on log-rank analysis but were not on multivariate analysis. It may be due to the small sample size, hence its insufficiency to detect the association between positive lymph node and DFS.

Although the patients received quite uniform treatments and had complete clinical data, the present study has many limitations. Firstly, it was a small-sized retrospective study from a single institution; hence, selection bias and imbalance are highly possible. Secondly, the follow-up period was short, and death events were too few to evaluate the association between TLCs and OS. Thirdly, some patients had only 3 or 4 times of weekly TLCs during CCRT, and TLC nadir may only be an approximate. Although the association between severe lymphopenia and poorer DFS was identified, causality cannot be established. Furthermore, TLCs may not adequately reflect immune function in patients with cancer.

## Conclusions

TLCs declined during CCRT, and severe lymphopenia was associated with poorer DFS in patients with locally advanced rectal cancer receiving surgery and adjuvant CCRT. CCRT had a profound effect on TLCs; however, pre-S TLCs were also associated with severe lymphopenia. These conclusions should be verified in larger prospective studies.

## Data Availability

The data and materials in the current study are available from the corresponding author on reasonable request.

## References

[1] Bray F, Ferlay J, Soerjomataram I, Siegel RL, Torre LA, Jemal A (2018). Global cancer statistics 2018: GLOBOCAN estimates of incidence and mortality worldwide for 36 cancers in 185 countries. CA Cancer J. Clin..

[2] Tveit KM, Guldvog I, Hagen S, Trondsen E, Harbitz T, Nygaard K (1997). Randomized controlled trial of postoperative radiotherapy and short-term time-scheduled 5-fluorouracil against surgery alone in the treatment of Dukes B and C rectal cancer. Norwegian Adjuvant Rectal Cancer Project Group. Br. J. Surg..

[3] Sauer R, Becker H, Hohenberger W, Rodel C, Wittekind C, Fietkau R (2004). Preoperative versus postoperative chemoradiotherapy for rectal cancer. N. Engl. J. Med..

[4] Roh MS, Colangelo LH, O'Connell MJ, Yothers G, Deutsch M, Allegra CJ (2009). Preoperative multimodality therapy improves disease-free survival in patients with carcinoma of the rectum: NSABP R-03. J. Clin. Oncol..

[5] Ngwa W, Irabor OC, Schoenfeld JD, Hesser J, Demaria S, Formenti SC (2018). Using immunotherapy to boost the abscopal effect. Nat. Rev. Cancer.

[6] Ceze N, Thibault G, Goujon G, Viguier J, Watier H, Dorval E (2011). Pre-treatment lymphopenia as a prognostic biomarker in colorectal cancer patients receiving chemotherapy. Cancer Chemother. Pharmacol..

[7] Kitayama J, Yasuda K, Kawai K, Sunami E, Nagawa H (2011). Circulating lymphocyte is an important determinant of the effectiveness of preoperative radiotherapy in advanced rectal cancer. BMC Cancer.

[8] Heo J, Chun M, Noh OK, Oh YT, Suh KW, Park JE (2016). Sustaining blood lymphocyte count during preoperative chemoradiotherapy as a predictive marker for pathologic complete response in locally advanced rectal cancer. Cancer Res. Treat..

[9] Tang C, Liao Z, Gomez D, Levy L, Zhuang Y, Gebremichael RA (2014). Lymphopenia association with gross tumor volume and lung V5 and its effects on non-small cell lung cancer patient outcomes. Int. J. Radiat. Oncol. Biol. Phys..

[10] Davuluri R, Jiang W, Fang P, Xu C, Komaki R, Gomez DR (2017). Lymphocyte nadir and esophageal cancer survival outcomes after chemoradiation therapy. Int. J. Radiat. Oncol. Biol. Phys..

[11] Choi CH, Kang H, Kim WY, Kim TJ, Lee JW, Huh SJ (2008). Prognostic value of baseline lymphocyte count in cervical carcinoma treated with concurrent chemoradiation. Int. J. Radiat. Oncol. Biol. Phys..

[12] Peng LC, Milsom J, Garrett K, Nandakumar G, Coplowitz S, Parashar B (2014). Surveillance, epidemiology, and end results-based analysis of the impact of preoperative or postoperative radiotherapy on survival outcomes for T3N0 rectal cancer. Cancer Epidemiol..

[13] Cho O, Oh YT, Chun M, Noh OK, Lee HW (2016). Radiation-related lymphopenia as a new prognostic factor in limited-stage small cell lung cancer. Tumour Biol..

[14] Sumiya T, Ishikawa H, Hiroshima Y, Nakamura M, Murakami M, Mizumoto M (2021). The impact of lymphopenia during chemoradiotherapy using photons or protons on the clinical outcomes of esophageal cancer patients. J. Radiat. Res..

[15] Balmanoukian A, Ye X, Herman J, Laheru D, Grossman SA (2012). The association between treatment-related lymphopenia and survival in newly diagnosed patients with resected adenocarcinoma of the pancreas. Cancer Investig..

[16] Campian JL, Ye X, Brock M, Grossman SA (2013). Treatment-related lymphopenia in patients with stage III non-small-cell lung cancer. Cancer Investig..

[17] Wild AT, Ye X, Ellsworth SG, Smith JA, Narang AK, Garg T (2015). The association between chemoradiation-related lymphopenia and clinical outcomes in patients with locally advanced pancreatic adenocarcinoma. Am. J. Clin. Oncol..

[18] Campian JL, Sarai G, Ye X, Marur S, Grossman SA (2014). Association between severe treatment-related lymphopenia and progression-free survival in patients with newly diagnosed squamous cell head and neck cancer. Head Neck.

[19] Wild AT, Herman JM, Dholakia AS, Moningi S, Lu Y, Rosati LM (2016). Lymphocyte-sparing effect of stereotactic body radiation therapy in patients with unresectable pancreatic cancer. Int. J. Radiat. Oncol. Biol. Phys..

[20] Hari DM, Leung AM, Lee JH, Sim MS, Vuong B, Chiu CG (2013). AJCC cancer staging manual 7th edition criteria for colon cancer: Do the complex modifications improve prognostic assessment?. J. Am. Coll. Surg..

